# A Parsimonious, Accurate, Predictive Model for Avian Egg Geometry

**DOI:** 10.3390/biology15151294

**Published:** 2026-08-04

**Authors:** Stefan T. Orszulik

**Affiliations:** Independent Researcher, Grove, Wantage OX12 0QA, UK; s.orszulik@ntlworld.com

**Keywords:** oval, egg shapes, egg geometry, pyriform eggs, predictive model, avian eggs

## Abstract

Over the years, many attempts have been made to mathematically describe an ovoid, both from a purely mathematical standpoint as well as to inform the underlying biological process of egg formation. Most existing formulations are complex and non-predictive, requiring each individual shape to be fitted separately. The aim of this study was to develop a parsimonious, elegant, and predictive model for the full range of avian egg geometry—one based on simple geometric components and a minimal number of parameters. This approach constructs an ovoid from two ellipses and a straight line, governed by only three adjustable parameters. The resulting model is predictive: it can accurately reconstruct the full contour of an avian egg using only the coordinate of maximum breadth and the breadth at one-quarter of the egg’s length from the pointed end. The mean error of this method is 0.047 (RMSE), which is competitive with other comparable methods. This accuracy arises because the geometric invariance of the model closely matches the allometric invariance observed in real avian eggs. Moreover, the ovoids generated by this method mostly conform closely to the Main Axiom, derived from Fermat’s extremum theory, making the model particularly suitable for engineers, designers, and ornithologists who require a straightforward means of constructing realistic ovoid shapes.

## Simple Summary

Understanding why bird eggs share certain characteristic shapes is a long-standing question in theoretical biology. Many mathematical descriptions of egg contours exist, but most are complex and must be individually fitted to each egg, limiting their biological generality. This study presents a simple geometric model that captures the essential features of the full range of avian egg shapes using only two ellipses and a straight line. With easily measured values—the position of maximum breadth and the breadth at one-quarter of the egg’s length from the pointed end—the model can accurately reconstruct the entire egg outline. The strength of this approach lies in its parsimony and predictive power. The model reflects the allometric invariance observed across bird species, meaning that the same geometric rules apply widely without needing to adjust parameters for each egg. It also aligns closely with a key mathematical principle derived from Fermat’s extremum theory, offering a mathematical basis for egg shapes as found in nature. This work provides a biologically grounded and mathematically robust framework for studying egg morphology, with practical applications for biologists, engineers, and designers who require accurate and easily generated ovoid shapes.

## 1. Introduction

Over the years there have been many attempts to mathematically model the ovoid shape [1,2,3,4,5,6,7,8,9,10,11,12,13,14]. The approaches to this have been many and varied. Recent developments include a sine-based model [14]; a number of models derived from the Hügelschäffer equation [5,8]; the Preston–Biggins model [1,2], which typically requires four or five parameters; an improvement of a model originally described by Smart [9,15]; and the parametric Gielis model [12]. Each of these models is briefly described below.

Narushin’s “rational” sine model is given in Equations (1)–(3):

(1) $$y=\pm \frac{B}{2}\sin^{n}\left(\frac{\pi (1-p)x}{x-pL}\right),$$

in which

(2) $$p=\frac{1}{2}\left[1+\frac{1}{2}{\left(\frac{w}{L}\right)}^{-1}\right],$$

(3) $$n=\frac{\ln\left(\frac{D_{p}}{B}\right)}{\ln\left[\sin\left(\pi \frac{1-p}{1-4p}\right)\right]}.$$

This model requires only four measurements (*L*, *B*, *w*, and *D_p_*): length, maximum breadth, distance of the maximum breadth from the y-axis, and diameter at a point *L*/4 from its pointed end. As can be seen, it is based on the sine and ln functions, and provides a good predictive model for the complete range of egg profiles.

Narushin and colleagues have also published several articles presenting predictive models derived from the Hügelschäffer equation [5,8]. The earlier paper [8] attracted worldwide attention [16,17,18,19] as a notable advance in the field, although the resulting contour did not appear visually satisfactory; this issue was subsequently addressed and improved in their later work [5].

The Preston model [4,10] represents one of the earliest attempts to provide a universal mathematical description of avian egg contours. Building on Preston’s original curvature-based formulation, Biggins [2] introduced a refined structure that expresses the profile as a smooth, continuous curve governed by up to five adjustable parameters: egg length, the position of maximum breadth (two parameters), a curvature parameter controlling blunt-end roundness, and a taper parameter determining the pointed-end profile. Together, these parameters allow the model to reproduce a wide range of egg morphologies, from nearly spherical forms to strongly pyriform outlines. Although computationally heavier than later compact models, the Preston–Biggins formulation remains influential because it captures the full contour with high descriptive accuracy and provides a mathematically coherent link between local curvature and overall egg shape. A further evaluation of the Preston–Biggins model was undertaken by Narushin [13] examining some of the advantages and disadvantages of the technique.

Smart’s egg shape formulation is grounded in the physiology of egg formation [9]. His model requires measurement of five parameters: egg length (*L*), maximum breadth (*B*), the displacement of the central axis at the level of maximum breadth (*w*), and the radii of the profile at the pointed and blunt ends. A practical evaluation of three egg-shape categories demonstrated that this five-parameter model reproduces their contours with high accuracy, outperforming other contemporary approaches. Narushin later conducted a more detailed assessment of Smart’s model [15], further clarifying its strengths and limitations.

The Gielis formulation adapts the superellipse to generate smooth, closed curves, capable of representing a wide spectrum of biological shapes [12]. This was applied specifically to avian eggs, showing that appropriate choices of the formula’s exponents and scaling parameters can reproduce the transition from blunt to pointed ends with high accuracy. In practice, the model uses three or four parameters to generate a continuous contour that accommodates spherical, elliptical, ovoid, and pyriform egg types. However, the limitations in the method lie in it being a parametric technique in which the parameters need to be fitted to each egg profile, as is discussed below.

In contrast to the approaches outlined above, the aim of the present study is to develop a model that is predictive, with minimal parameters, and constructed entirely from basic geometric shapes. Although many existing methods incorporate geometric principles, they do not rely on truly fundamental forms. Because the geometry of avian eggs appears to lie on a natural continuum—from circle to ellipse to ovoid—the goal here was to generate the egg contour using elementary shapes in a manner that is both mathematically elegant and biologically meaningful. The rationale for this approach is straightforward: a model built from simple, universal geometric elements that captures the essential features of egg morphology whilst remaining meaningful, efficient, and broadly applicable.

Predictive Power: There are several main types of mathematical methods used to model egg outlines. In some cases, a mathematical equation is presented which needs to be fitted to each egg shape. Whilst interesting, such an approach is of limited use, requiring multiple measurements of many egg contours, and cannot be described as universal. Some equations are x–y-type Cartesian, which are the easiest to apply, whilst others are parametric equations. Parametric equations are often considered more difficult than Cartesian equations because they introduce an extra variable and require multi-step processes to analyse and interpret. Significant manipulation is needed to translate Cartesian coordinates of an egg shape to fit the parametric equation. An example of this is the parametric equation presented by Gielis [12] (Equation (4)), which is not only parametric but also requires parameter estimation to be fitted to each individual egg profile using some form of optimisation technique.

(4) $$r\;(\varphi )=a({{\left|\cos\left(\varphi /4\right)\right|}^{n_{2}}+\left|{sin(\varphi ∕4)|}^{n_{2}}\right)}^{-1/n_{1}}$$

where *r* is the radius at the polar angle *φ*, and *a*, *n*_1_, and *n*_2_ are parameters to be estimated.

The most useful (and difficult to formulate) are the predictive equations, whereby a number of egg measurements are fed into an equation which is able to produce the entire egg contour. The Preston–Biggins (PB) formula [2] produces an accurate predictive description of an egg contour, which may be expected as it uses five measured parameters to generate the egg shape. The Narushin model [5] (hereafter NORG, Equation (5)) is a predictive method using just the length (*L*), the position of the maximum breadth (*B* and *w*) and the breadth one-quarter of the length from the pointed end (*D_p_*).

However, the PB approach does not conform to the Main Axiom as defined by Narushin [13], which states that the extremes of the model and egg (positions of the length and maximum breadth) should match. The Main Axiom is based on Fermat’s Interior Extremum Theory [20]. To explain the importance of the Main Axiom, we need to consider its biological and mathematical implications—since any model would not be expected to match the entire contour of an egg exactly, these are regarded as the most important positions (length and breadth) in terms of biology (the width as the egg travels down the oviduct) and mathematics (the egg shape fitting into a defined space). Similarly, the Gielis method cannot claim to conform to the Main Axiom. The NORG model does conform and, in his respect, can be regarded as a superior method.

(5) $$Y=\pm \frac{B}{2}\left[\frac{1}{\sqrt{1+4\frac{(x+w{)}^{2}}{L^{2}-4x^{2}}}}+\left(\sqrt{\frac{1+4\frac{(w-w_{p}{)}^{2}}{L^{2}-4w^{2}}}{1+4\frac{(x+w_{p}{)}^{2}}{L^{2}-4x^{2}}}}-\frac{1}{\sqrt{1+4\frac{(x+w{)}^{2}}{L^{2}-4x^{2}}}}\right)\left(1+\frac{x+w}{\sqrt{(x+w{)}^{2}}}\right)\left(\frac{x}{L}+\frac{1}{4}\right)\right]$$

There are many other models available, each with their own advantages and disadvantages, but for the purposes of this article, the predictive models are regarded as the most important. One recent and significant predictive model is the CE (Circle–Ellipse) model [21], which is entirely based on basic geometric shapes—in this case a circle, an ellipse, and a straight line. This model is extremely minimal, having just two adjustable parameters (one from the ellipse and one from the straight line, the diameter of the circle being fixed to the length of the egg). Following regression analysis of the data, the CE model is able to predict the ovoid contour from just the position of the maximum breadth. Generally, the minimum number of adjustable parameters required to produce an accurate egg model is considered to be three—preferably the position of the maximum breadth (*x* and *y* coordinate) and another point, usually one-quarter of the length from the pointed end (*D_p_*). After all, the circle has one variable (the radius) and the ellipse has two (minor and major axes), and hence an egg shape would be expected to have at least three adjustable parameters, probably more. Since the CE model has just the two parameters, this seems insufficient to accurately describe an egg shape. The explanation for this lies in the geometric invariance of the model aligning with the allometric invariance of avian eggs. These and other terms need further explanation:

Geometric and Allometric Invariance: When considering geometric invariance, it is instructive to consider the transition from a circle to an ellipse through to an egg shape. A circle has just one variable, the radius. Once the radius has been set, there are no possible variations. The ellipse has two variables, the major and minor axes (Equation (6)). However, there are many ellipse-looking shapes that don’t necessarily conform with Equation (6), and indeed this applies to some elliptical egg shapes. Thus, these shapes are visually ellipses but mathematically not “pure” ellipses. This describes the concept of “geometric invariance”—mathematically an ellipse needs to comply with Equation (6), but there can be many shapes that look like an ellipse but do not comply fully with Equation (6).

The same concept applies to egg contours. There are many egg shapes in nature—there is variation in shape within a species and a much greater variation between species. Nevertheless, avian eggs display a degree of invariance—allometric invariance [22]. A consequence of this is that the entire contour can be predicted with reasonable accuracy just from the coordinate of the widest point. This is contrary to expectations where it is thought that the minimum is three datapoints on which to build a predictive model, but this feature very much depends on the model. The geometric invariance of the CE model matches closely with the allometric invariance of avian eggs, suggesting that nature follows the mathematics of the CE model.

Parsimony: It is always possible to add more and more terms to an equation to improve the fit, but such an approach is not generally accepted since each term loses its meaning and each added term is likely to become less significant in terms of *p*-value. The aim is to describe a particular shape in the minimum of terms, preferably with each term having some real meaning rather than just improving the fit.

If we consider the Gielis formula, there are three parameters that need to be estimated for each shape (*a*, *n*_1_, and *n*_2_—Equation (4)), and these have little direct meaning except as mathematical terms. The predictive NORG model has four terms (*L*, the position of widest part—*D* and *w*, and *D_p_*. Each of these parameters has real meaning.

Geometric Shapes: Visually, there is a progression of shapes from circle and ellipse to an egg shape. However, finding a purely geometric equation to an egg shape has proven difficult, and hence other solutions have been found, such as Gielis’s parametric equation and the NORG equation, which is based on the Hügelschaffer model. The CE model is a purely geometric model that accurately predicts the avian egg contour. The purpose of this current research is to build on the CE model to create a more accurate model that is both minimal and meaningful.

## 2. Materials and Methods

### 2.1. Strategy of the Current Work

To build a minimal, accurate, predictive, geometric-based model along the lines of the CE model, following the principles outlined above, it was decided that such a predictive model should be based on the same datapoints as the NORG model i.e., the maximum breadth coordinates (*D*, *w*) and the datapoint *D_p_*. In order to accomplish this, the model would need three adjustable parameters. Therefore, the CE model was modified to use two ellipses and a straight line. Each ellipse has one adjustable parameter (the minor axis, since the major axis is fixed), and the third adjustable parameter is the slope of the straight line. The model would then be fitted to a range of avian eggs, providing data on the three adjustable parameters and the egg dimensions *w*, *D*, and *D_p_*. These data could then be subjected to regression analysis with the aim of developing a predictive model.

### 2.2. Model Theory

The method described in this article involves the fusion of two ellipses controlled by a straight line (henceforth known as the EE model). The formulas for the two ellipses are as follows:

(6) $$f_{1}=\;\pm b_{1}\sqrt{(1-(x^{2}/a_{1}^{2})}$$

(7) $$f_{2}=\pm b_{2}\sqrt{(1-(x^{2}/a_{2}^{2})}$$

where *a* and *b* are the semi-major and semi-minor axes, respectively.

The equation of a straight line is:

(8) $$f_{3}=mx+\;c$$

The egg contour is generated from these basic geometric shapes using the following equation:

(9) $$f_{4}=\;f_{1}\left(1-f_{3}\right)+\;f_{2}f_{3}$$

For a better comparison with previous work in this area [5,8,14], the length of the eggs is standardised to 5.7, in line with Romanoff and Romanoff [23], with the proper recalculation of all other measured parameters. Thus, with the egg centred on the point (0, 0), all egg measurements are rescaled such that the length falls between −2.85 and +2.85. Therefore, *a*_1_ and *a*_2_ are set at 2.85. The constant term in Equation (8) is fixed at 0.5, and so the only parameters that are adjustable are *b*_1_ and *b*_2_ (the breadths of the two ellipses) and *m* (the slope of the straight line). The value of *c* = 0.5 has been chosen as this is the value of *c* when the straight line lies at the points *y* = 0 and +1 at the boundaries of the design space (*x* = −2.85 and +2.85, respectively).

**Figure 1:**
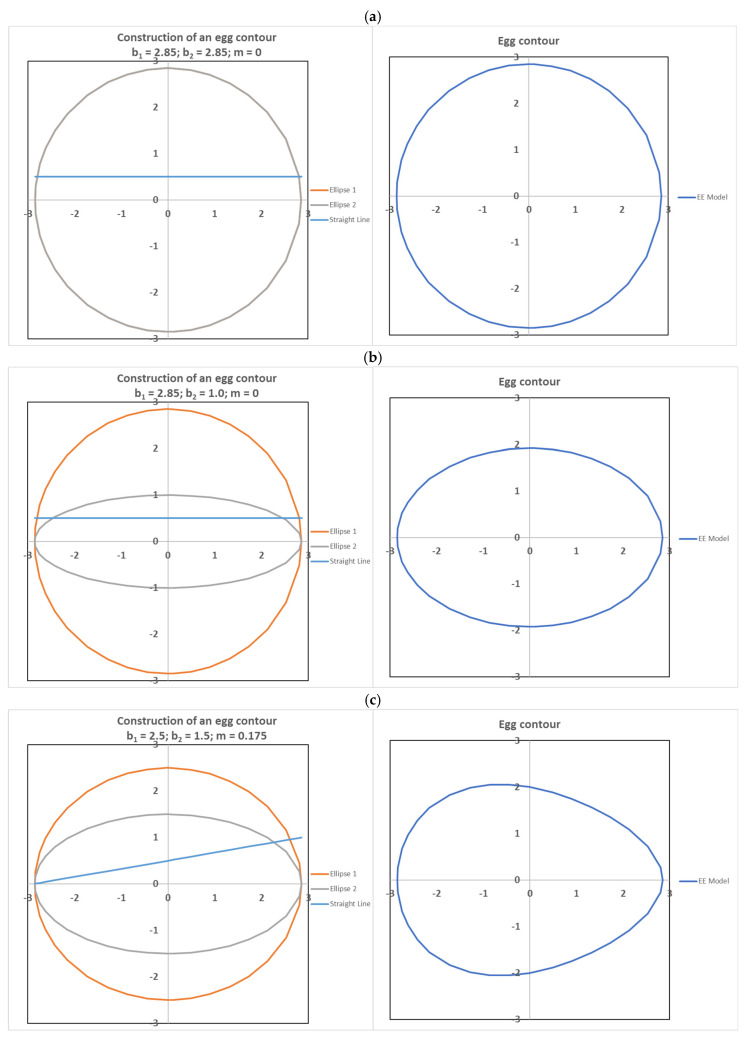

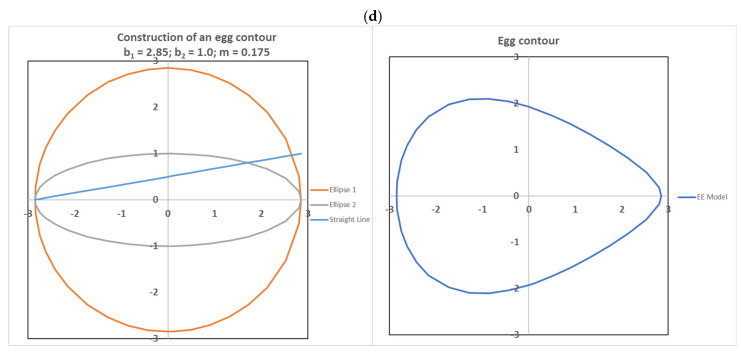
Use of basic geometric shapes to generate a full range of avian egg contours: (**a**) circular, (**b**) elliptical, (**c**) oval, (**d**) pyriform.

Nishiyama [7] has suggested that there are four basic avian egg shapes: circular, elliptical, ovoid, and pyriform (pear shaped). Figure 1 shows that the basic geometric shapes embedded in Equation (9) are able to generate the full range of egg shapes by varying the levels of just the three parameters, *b*_1_, *b*_2_ and *m*.

Having generated a geometric-based equation that encompasses all egg shape types (Equation (9)), the next task is to fit this equation to a full range of avian eggs. This was accomplished using least squares analysis, which provides a fit between actual and model data. These data are then used to produce a predictive model, where the values of *a*_1_, *a*_2_, and *m* are computed from egg parameters *w*, *D*, and *D_p_*. This is fully discussed in the following section.

## 3. Results and Discussion

A wide range of avian eggs was fitted to the EE model (Equation (9)) with the aid of the optimisation tool available in MS Excel (Solver add-in), in which the RMSE (Root Mean Square Error) of each egg model was minimised. The results are given in Table 1, in which the EE model is compared to the other geometric-based model (CE) and the Gielis model. The Gielis model was chosen for comparison since it is a recently published method, is not a predictive model, and also uses an optimisation procedure to fit the model to egg contours. In keeping with these [7,21] and other articles on this topic, RMSE is used as the main measure of error in the analysis presented in Table 1.

**Figure 2:**
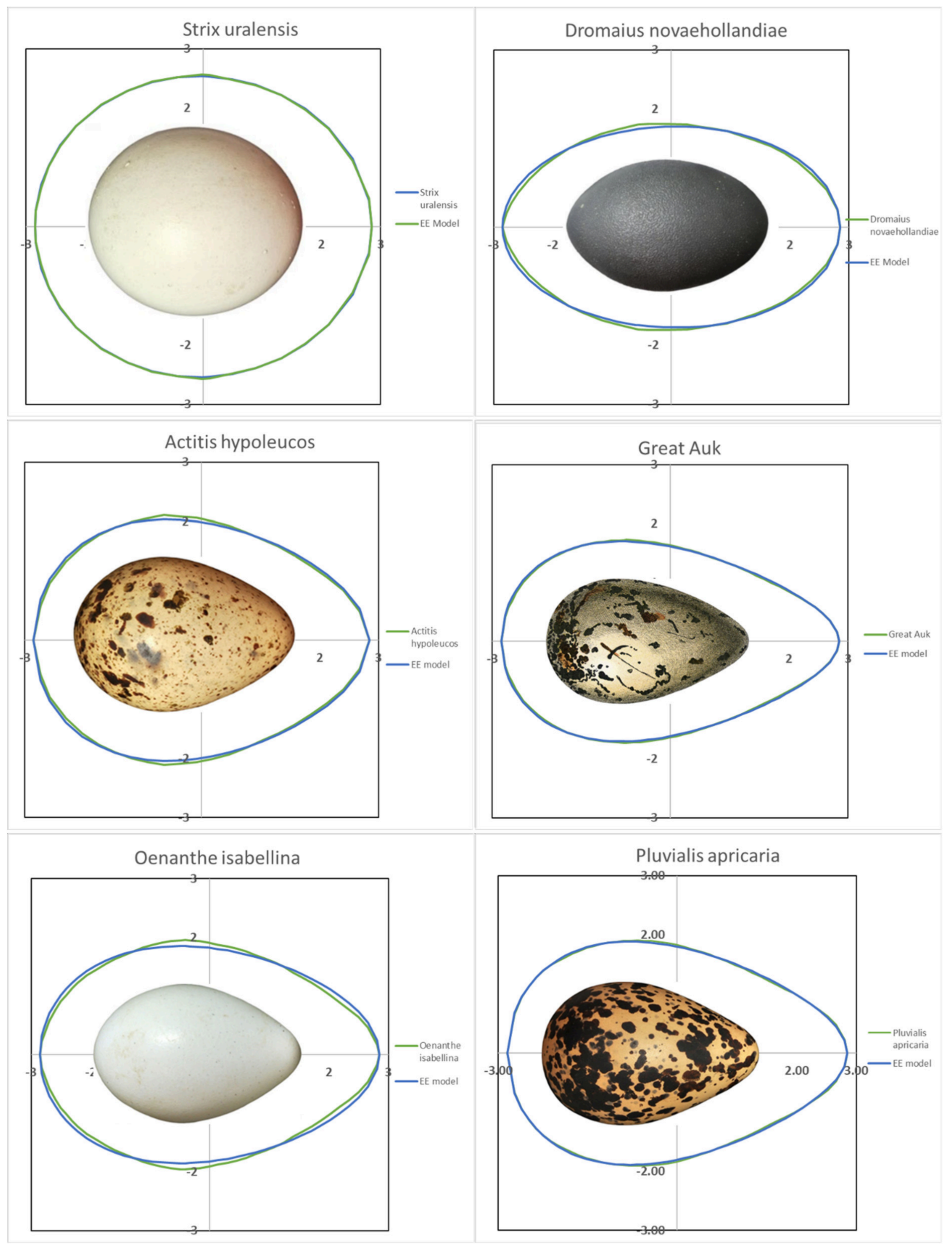
Selected examples of the EE Model fitted to a range of avian egg shapes. Images obtained from websites cited, ref. [8].

Figure 2 shows the EE model fitted to a range of avian eggs.

The data in Table 1 demonstrates that all three models provide good fits for real avian eggs. However, it should be noted that the CE model has two parameters, whereas the Gielis and EE models have three. Furthermore, the Gielis model is parametric, making it more difficult to apply, and the adjustable parameters in the Gielis model have little obvious meaning. The CE and EE models give very similar results, which is perhaps not surprising since they are both based on geometric shapes and employ a similar methodology. However, the EE model has one more parameter, and hence this enables further statistical analysis, leading to an efficient and accurate predictive model.

Data from the EE model over the full range of eggs shown in Table 1 (*b*_1_, *b*_2_, and *m*) was collated with the corresponding egg dimensions (*w*, *D*, and *D_p_*). Regression analysis using design surface analysis methodology [24] provided predictive equations for *b*_1_, *b*_2_, and *m*. Following the removal of less significant factors, the final model is shown in Table 2.

Using the coefficients presented in Table 2, it is possible to predict values of *b*_1_, *b*_2_, and *m* for any egg from just three egg measurements, *w*, *D*, and *D_p_*. Using a much-expanded selection of avian eggs, these predicted values along with original values are given in Table 3.

Data in Table 3 suggests that excellent predictive egg contours can be generated using Equation (9) and the values of *b*_1_, *b*_2_, and *m*, as calculated according to Table 2. Table 3 also includes the predictions for several additional egg contours to demonstrate the methods universal application.

**Figure 3:**
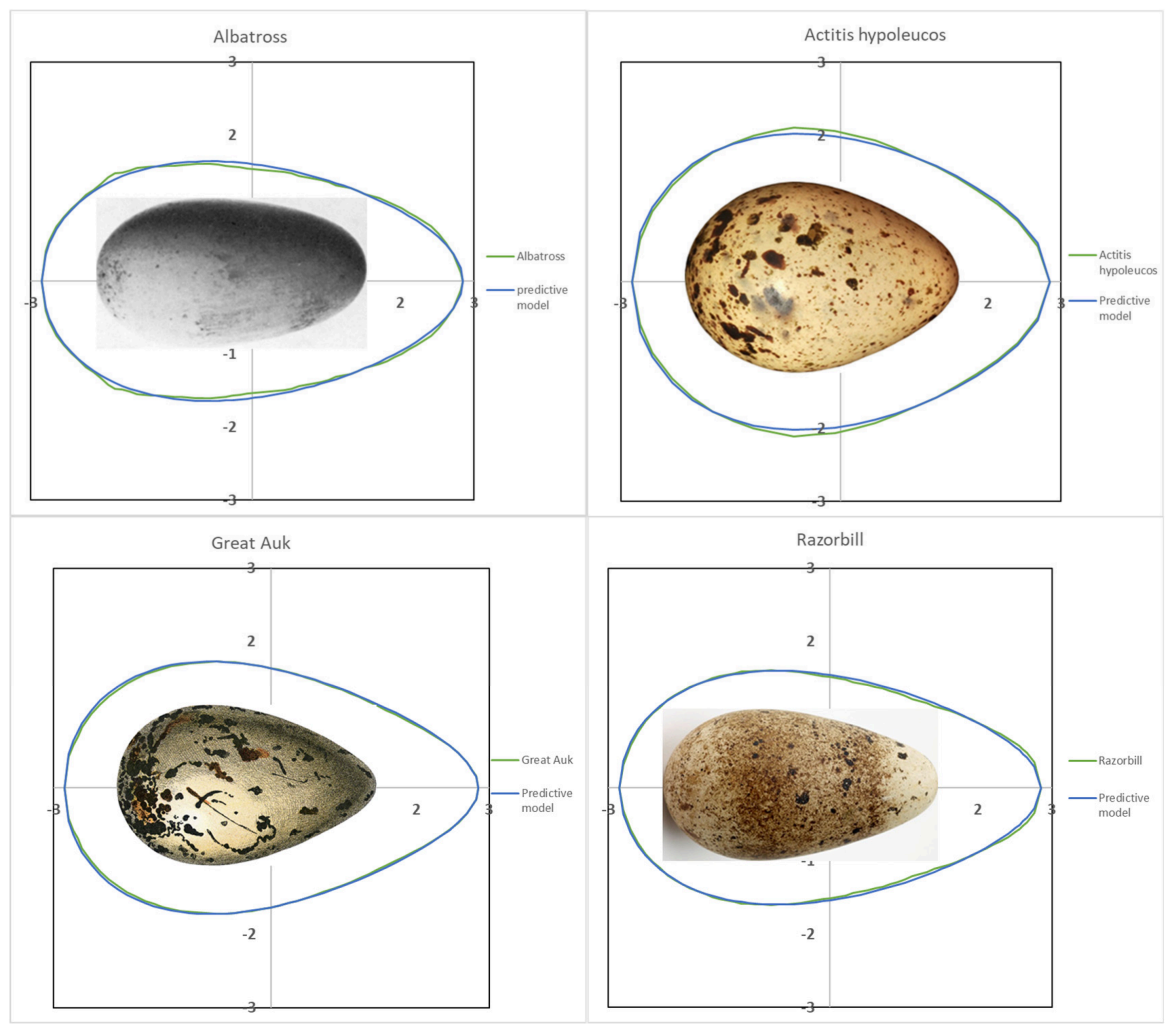
Selected examples of the EE predictive model fitted to a range of avian egg shapes. Images obtained from websites cited, ref. [8].

In order to visualise these results, a selection of actual and predicted egg contours is shown in Figure 3.

To judge the usefulness of the EE predictive model, it is instructive to compare it to another predictive model with the same number of inputs. The NORG model is just such a model using the exact same inputs—the position of the widest point and the width one-quarter of the length from the pointed end. Other models, such as the Preston–Biggins model, may also produce good fits, but this is accomplished through the use of more egg measurements, making them less useful. Furthermore, the Preston–Biggins model does not comply with the Main Axiom [13]. The NORG model does comply with the Main Axiom, and indeed the EE predictive model also closely conforms to this axiom, as it uses the position of maximum breadth in modelling the egg contour. A comparison of the errors (RMSE and R^2^) of the predictive EE and NORG models over the extended range of avian eggs is shown in Table 4.

As can be seen from Table 4, the errors in the predictive EE model are, on average, slightly lower than those of the NORG model. There is no correlation between models’ performance and egg shape fundamentals (elongation, ovoidness, and asymmetry ratio—*quod vide*). Overall, both models perform well considering that only three adjustables have been employed—this can be attributed to the geometric invariance in the models being aligned with the allometric invariance of avian eggs, in a similar manner to that of the CE model. It is proposed that the predictive power of the EE model, its close conformity to the Main Axiom combined with the minimal requirement of just three real egg measurements, makes it a model of choice; in short, it is parsimonious and elegant, and closely mimics the invariance of avian eggs found in nature.

### 3.1. Physiological Parameter Interpretations

As discussed above, the parameters *b*_1_, *b*_2_, and *m* in the EE method have real mathematical meaning, as they refer to the breadths of the ellipses and the slope of the straight line. The question arises as to how these parameters relate to the final egg shape in a biologically meaningful way.

General descriptive terms such as round, elliptical, ovoid, and pyriform have long been used to classify egg shapes, but a range of quantitative metrics has also been proposed. These measures are considered biologically informative because they are thought to reflect both the evolutionary diversification of egg forms and to inform on the physical pressures acting on the developing egg as it moves through the oviduct. These quantitative measures include elongation, ellipticity, ovoidness, asymmetry ratio, pointedness, pear-shapedness, and others [1]. Each of these has its own formula. Probably the most useful measures are elongation, ovoidness, and asymmetry ratio, the equations for which are shown in (10)–(12).

*Elongation* = *B*/*L* (10)

*Ovoidness* = *P*/(*L* − *P*) (11)

*Asymmetry Ratio* = *P*/*L* (12)

where *P* = *w* + *0.5L*.

The correlation between the parameters *b*_1_, *b*_2_, and *m* and the egg measures elongation, ovoidness, and asymmetry ratio was examined, as shown in Table 5.

Interestingly, as shown in Table 5, *b*_1_ is the most significant parameter for elongation, but is not significant for ovoidness and asymmetry ratio. The parameters *b*_2_ and *m* have a significant influence on all the egg measures. Further work will need to be undertaken to fully understand the implications of these correlations, but these results suggest that some egg types may be adequately described with the use of just two parameters, whilst others will require all three, depending on their shape.

The EE method provides a clear mathematical description of egg geometry which can be the basis for future automated, image-based extraction of egg parameters. Since the EE model expresses the egg contour using a small number of biologically meaningful parameters, it may be readily integrated into modern computer-vision methodologies. Its low-dimensional parameterisation makes it well suited for modern morphometric and computer-vision approaches, enabling rapid extraction of key traits and facilitating large-scale, standardised analyses across diverse datasets.

### 3.2. Limitations of the EE Method and Future Work

The aim of achieving an egg-shaped model based solely of geometric basics has been achieved, but only by going through a regression procedure. The NORG model rests entirely on mathematical principles and, though complex, achieves a level of conceptual purity that distinguishes it from the EE and other approaches. It would be desirable to develop an EE model that does not rely on a regression step, and this will be the focus of future work.

The EE method has been applied across the principal avian egg morphologies (circular, elliptical, ovoid, and pyriform). However, some natural egg forms fall close to—but not fully within—these categories. Extending validation to include such additional shapes would therefore be valuable.

## 4. Conclusions

The egg shape found in nature could be considered to be a continuum of geometric shapes from a straight line, to a circle, to an ellipse, to an ovoid. However, despite many years of research, most mathematical descriptions of egg shapes are mathematically very complex and do not appear to be closely associated with basic geometric shapes. The purpose of this project was to develop a predictive mathematical description of ovoid shapes found in nature based solely on basic geometric shapes. There were further constraints to the work in that the model should be minimalist (as few input parameters as possible); it should be elegant (using easily understandable parameters); should comply closely to the Main Axiom; should be predictive based on egg measurements; and, of course, should produce predicted egg contours that closely match those of the natural system.

The approach taken was to build a model along the lines of the CE model but with one more parameter, thereby increasing the degrees of freedom and enabling a more accurate model to be built. The extra parameter was created by using two ellipses, rather than a circle and an ellipse as in the CE model. This produced a model whose results were similar to that of the CE model, but the extra parameter of the EE model enabled a deeper regression analysis to be performed. At this stage the CE and EE models were compared to the Gielis model—all three performed well, but the Gielis model is a parametric method that needs to be fitted to each egg contour, making it less useful.

Having collected data on the performance of the EE model, regression analysis was performed which successfully resulted in prediction for the three model parameters (*b*_1_, *b*_2_ and *m*) in terms of the three egg parameters (*w*, *D*, and *D_p_*). This predictive EE model is able to accurately create the outlines of all types of egg shapes defined by Nishiyama [7]: circular, elliptical, ovoid, and pyriform. Comparison of this new EE predictive model with the NORG model, which uses the exact same input parameters, showed the EE model to be overall slightly more accurate. Furthermore, the EE model conforms closely to the Main Axiom. In summary, the EE model is based on basic geometric shapes, and is minimalist and accurate. Its ease of use should enable practical applications for biologists, engineers, and designers who require accurate and easily generated ovoid shapes.

## Figures and Tables

**Table 1:** Comparison of three models, as measured by their Root Mean Square Errors, for a range of avian eggs *.

	Gielis	CE Method	EE Method
*Actitis hypoleucos*	0.026	0.045	0.0453
*Dromaius novaehollandiae*	0.061	0.059	0.0589
Great Auk	0.028	0.021	0.0202
Great Snipe	0.031	0.059	0.0589
Guillamot	0.031	0.042	0.0427
*Limosa limosa*	0.046	0.022	0.0216
*Numenius phaeopus*	0.031	0.045	0.0448
*Oenanthe isabellina*	0.064	0.079	0.0792
*Pluvialis apricaria*	0.048	0.02	0.0198
Razorbill	0.038	0.03	0.0296
*Stercorarius skua*	0.022	0.035	0.0350
*Strix uralensis*	0.029	0.022	0.0216
*Tringa totanus*	0.064	0.025	0.0247

* Images obtained from websites cited, ref. [8]. Coordinates obtained using standard software (e.g., MS Paint 11.2605.71.0), centred and scaled.

**Table 2:** Results of regression analysis—parameters *b*_1_, *b*_2_, and *m* in terms of egg measurements.

Term	Coef for *b*_1_	Coef for *b*_2_	Coef for *m*
Constant	−8.15	2.739	0.152
*w*	−2.4	−0.082	0.0898
*D*	16.56	−8.898	0.5026
*D_p_*	−11.38	9.909	−0.623
*D* ^2^	2.78		0.264
*D_p_* ^2^	13.77	−5.944	0.7515
*w* × *D*	7.21	−3.226	0.2866
*w* × *D_p_*	−9.19	5.072	−0.5086
*D* × *D_p_*	−16.15	5.176	−0.9627

where *w* and *D* are the coordinates of the widest point, and *D_p_* is the width one-quarter of the length from the sharp end.

**Table 3:** Original and predicted values of egg parameters.

	Original Values			Predicted Values		
Species	*b*_1_	*b*_2_	*m*	*b*_1_	*b*_2_	*m*
*Actitis hypoleucos*	2.57	1.42	0.14	2.54	1.42	0.14
*Actitis macularius*	2.48	1.34	0.15	2.52	1.28	0.15
Albatross	1.96	1.23	0.17	1.97	1.22	0.16
*Charadrius morine*	2.31	1.29	0.15	2.31	1.27	0.15
*Dromaius* *novaehollandiae*	1.63	1.78	0.11	1.61	1.77	0.11
*Galerida cristata*	2.49	1.41	0.12	2.29	1.53	0.12
Great Auk	2.20	1.03	0.17	2.18	1.03	0.18
Great Snipe	2.50	1.31	0.15	2.50	1.30	0.15
Guillamot	2.23	1.12	0.17	2.19	1.14	0.17
King Penguin	2.67	1.47	0.11	2.38	1.62	0.11
*Larus atricilla*	2.32	1.28	0.14	2.23	1.33	0.15
*Limosa limosa*	2.47	1.20	0.16	2.42	1.21	0.16
*Numenius phaeopus*	2.47	1.19	0.17	2.47	1.19	0.17
*Oenanthe isabellina*	2.38	1.26	0.11	2.22	1.41	0.14
*Pluvialis apricaria*	2.43	1.17	0.16	2.40	1.17	0.17
Razorbill	1.98	1.08	0.17	1.97	1.06	0.17
*Stercorarius skua*	2.26	1.33	0.14	2.23	1.32	0.15
*Strix uralensis*	2.99	2.09	0.03	2.95	2.09	0.03
*Tringa totanus*	2.54	1.13	0.17	2.50	1.15	0.17

**Table 4:** Comparison of errors of predictive EE and NORG models of avian egg contours.

	NORG		Predictive EE	
	RMSE	R^2^	RMSE	R^2^
*Actitis hypoleucos*	0.0811	0.9902	0.0475	0.9992
*Actitis macularius*	0.0401	0.9960	0.0500	0.9989
Albatross	0.0259	0.9966	0.0410	0.9990
*Charadrius morine*	0.0861	0.9819	0.0354	0.9994
*Dromaius novaehollandiae*	0.0590	0.9955	0.0599	0.9978
*Galerida cristata*	0.0426	0.9954	0.0719	0.9985
Great Auk	0.0403	0.9964	0.0213	0.9997
Great Snipe	0.0910	0.9873	0.0592	0.9984
Guillamot	0.0544	0.9923	0.0460	0.9986
King Penguin	0.0386	0.9963	0.0913	0.9985
*Larus atricilla*	0.0640	0.9935	0.0649	0.9981
*Limosa limosa*	0.0201	0.9988	0.0301	0.9997
*Numenius phaeopus*	0.0447	0.9947	0.0450	0.9991
*Oenanthe isabellina*	0.0847	0.9870	0.0797	0.9969
*Pluvialis apricaria*	0.0208	0.9988	0.0255	0.9998
Razorbill	0.0449	0.9904	0.0321	0.9994
*Stercorarius skua*	0.0856	0.9748	0.0373	0.9994
*Strix uralensis*	0.0417	0.9986	0.0246	0.9999
*Tringa* *t* *otanus*	0.0584	0.9935	0.0291	0.9997

**Table 5:** Pearson correlation between parameters *b*_1_, *b*_2_, and *m* and the measures elongation, ovoidness, and asymmetry ratio.

		*b*_1_	*b*_2_	*m*
elongation	Pearson correlation	0.873	0.667	−0.748
	*p*-value	0.000	0.009	0.002
				
ovoidness	Pearson correlation	−0.113	−0.890	0.894
	*p*-value	0.701	0.000	0.000
				
Asymmetry Ratio	Pearson correlation	−0.133	−0.919	0.921
	*p*-value	0.651	0.000	0.000

## Data Availability

Available from the author on reasonable request.

## Abbreviations

The following abbreviations are used in this manuscript:

*r*	Radius
*φ*	Polar angle
*a*, *n*_1_, and *n*_2_	Parameters to be estimated
*B*	Oval maximum breadth
*w*	Distance of the maximum breadth of the oval from the y-axis
*D_p_*	Diameter of the oval at a point *L*/4 from its pointed end
*L*	Oval length
RMSE	Root Mean Square Error—$RMSE=\;\sqrt{\frac{\sum_{i=1}^{n}({{Predicted}_{i}-{Actual}_{i})}^{2}}{n}}$
R^2^	Coefficient of determination $R^{2}=\frac{{\left(yi-\hat{y}\right)}^{2}}{{\left(yi-\bar{y}\right)}^{2}}$
CE	Circle–Ellipse model [21]
EE	Ellipse–Ellipse model reported herein
NORG	Model reported in [5]
PB	Preston–Biggins model
